# Prevalence of irritable bowel syndrome and metabolic syndrome among young adults in an annual health check‐up setting

**DOI:** 10.1002/jgh3.12639

**Published:** 2021-09-09

**Authors:** Narendra S Javadekar, Gauri A Oka, Ashwini S Joshi, Parag Vaste, Sandeep Tamane, Parimal S Lawate

**Affiliations:** ^1^ Department of Medicine Deenanath Mangeshkar Hospital and Research Centre Pune India; ^2^ Department of Research Deenanath Mangeshkar Hospital and Research Centre Pune India; ^3^ Department of Gastroenterology Deenanath Mangeshkar Hospital and Research Centre Pune India

**Keywords:** irritable bowel syndrome, metabolic syndrome, Rome III

## Abstract

**Background and Aim:**

Some studies have found a positive association between irritable bowel syndrome (IBS) and metabolic syndrome; however, none are from India.

**Methods:**

We conducted a cross‐sectional study of 1040 adults aged between 18 and 50 years. Individuals from the annual health check‐up setting were screened using anthropometry and biochemistry. Based on the results, they were identified as with and without metabolic syndrome. We excluded individuals who were already diagnosed with metabolic syndrome or those who were already on medication for diabetes mellitus or hypertension or dyslipidemia. All the participants were administered the Rome III questionnaire for the diagnosis of IBS.

**Results:**

Metabolic syndrome was found in 307 of 1040 (29.5%) while 33 of 1040 (3.2%) had IBS. The proportion of IBS was not significantly different between participants with and without metabolic syndrome (1.6% *vs* 3.8% respectively; *P* = 0.06). Those with IBS had significantly greater mean weight (72.4 *vs* 67.2 kg; *P* = 0.009), mean waist circumference (88.8 *vs* 85.2 cm; *P* = 0.011), mean body mass index (BMI) (26.2 *vs* 24.2 kg/m^2^; *P* = 0.002), and higher mean fasting glucose (96 *vs* 89 mg/dL; *P* < 0.000) respectively.

**Conclusion:**

The prevalence of metabolic syndrome and IBS are comparable to previous literature from India. There was no association between metabolic syndrome and IBS.

## Graphical abstract

The proportion of irritable bowel syndrome (IBS) was not significantly different between participants with and without metabolic syndrome. We found higher mean waist circumference and fasting glucose values and lower mean HDLC values in participants with IBS. The mean weight and mean BMI values were also found to be significantly higher in the IBS group.

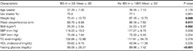

## Introduction

Irritable bowel syndrome (IBS) is one of the most common disorders among the spectrum of functional gastrointestinal (GI) disorders, especially in advanced countries. It affects almost 20% of the population causing a significant functional and socioeconomic burden.^1^ An altered GI motility due to psychosocial stress, changes in the gut microflora, intestinal inflammation, and dietary factors are thought to be crucial in its pathogenesis.^2^ The development of Rome III criteria (2006) revolutionized the approach to functional bowel disorders, including IBS, by bringing uniformity and standardization in the diagnosis and reporting of these conditions. The prevalence of IBS in India, ranging between 4.2% and 7.9%,^3^, ^4^ is certainly less as compared to that in the developed countries. This may be due to dietary and cultural factors^5^ and in part, to the sensitivity of the criteria used for the diagnosis.

Metabolic syndrome, a conglomeration of risk factors (dysglycemia, increased blood pressure, dyslipidemia, and central obesity), is said to be the precursor of cardiometabolic disorders such as diabetes mellitus and coronary artery disease.^6^, ^7^ Studies conducted across urban populations in India have found metabolic syndrome to be fairly common, afflicting between 26% and 31.6% of the adult population. The relationship between metabolic syndrome and functional bowel disorders has come under the scanner of researchers with the evolution of the concept of the gut–brain axis as the common linkage between the two. The gut became the focus of researchers interested in unraveling the key to the perplexity of cardiometabolic disorders due to its extensive neuroendocrine and immune functions and the evolving knowledge about gut microbiome and epigenetics. Published literature reports associations between functional GI disorders and components of metabolic syndrome or metabolic syndrome itself.^1^, ^8^, ^9^, ^10^, ^11^, ^12^, ^13^, ^14^ Although a positive association between gastroesophageal reflux and metabolic syndrome can be easily explained by abdominal adiposity, IBS defies such an explanation.^15^

A population‐based study of 1096 participants in Japan using the Rome III questionnaire found that IBS was indeed positively associated with metabolic syndrome, even after adjusting for all the confounding factors such as age, sex, BMI, and physical activity. A case–control study from South Korea reported a significantly higher prevalence of metabolic syndrome in patients with IBS than those without IBS using the Rome III criteria.^16^ In a study on individuals diagnosed with non‐alcoholic fatty liver disease (NAFLD) attending an outpatient clinic in coastal India, Singh et al. found a strong association between IBS and the components of metabolic syndrome, although they did not use the Rome questionnaire for the diagnosis of IBS.^17^

Given the abovementioned studies and the possible etiopathogenic association between IBS and metabolic syndrome, the present study was carried out to determine the prevalence of metabolic syndrome and IBS (using Rome III criteria) and the association, if any, between the two.

## Material and methods

This cross‐sectional observational study was carried out between 2015 and 2017 in the executive annual check‐up outpatient department of a multispecialty tertiary care hospital. The study was approved by the Institutional Ethics Committee (Approval number: 2015_Aug_NJ_174). The sample size was calculated based on the prevalence of metabolic syndrome. Assuming a 20% prevalence of metabolic syndrome in the general population with ±4% marginal error in the estimation at 5% level of significance and 90% power, the sample size was estimated as 1040. The Rome III questionnaire uses symptom‐based criteria for the diagnosis of IBS. Symptom‐based diagnosis of IBS in the absence of red flag symptoms is acceptable for younger individuals, while a colonoscopy is recommended in patients over 50 years of age.^18^ So, we decided to include subjects only up to 50 years of age. We excluded individuals who were already diagnosed with metabolic syndrome or those who were already on medication for diabetes mellitus or hypertension or dyslipidemia. This was done to exclude the potential influence of medications and changes in diet and lifestyle after treatment initiation on the gut symptoms.^6^ Thus, 1040 consecutive adults between the age group of 18 and 50 years coming for yearly health check‐ups and who fulfilled the screening criteria were enrolled during the study period. After obtaining informed consent, all the participants completed the self‐administered Rome III questionnaire (either English or Rome foundation‐approved Marathi translated version). For the diagnosis of metabolic syndrome, clinical examination (height, weight, waist circumference, blood pressure, and systemic examination) and laboratory investigations (lipid profile and blood sugar levels) were done. Height was measured with a calibrated stadiometer accurate up to 0.1 cm. The weight was measured without footwear and extra clothing using a digital weighing scale with an accuracy of 0.01 kg. The waist circumference was measured using non‐extensible tape and was measured at the smallest diameter between the lower border of the 12th rib and the iliac crest. Blood pressure was measured using a calibrated sphygmomanometer.

A participant was considered to have metabolic syndrome upon fulfillment of three or more criteria out of the following: increased waist circumference (males >90 cm and females >80 cm), increased blood pressure (≥130/85 mm Hg), elevated triglycerides (>150 mg/dL), elevated fasting glucose (≥100 mg/dL), and decreased HDL cholesterol (males <40 mg/dL and females <50 mg/dL). The remaining participants were classified as having “no metabolic syndrome.” IBS was diagnosed using the Rome III questionnaire if a participant had recurrent abdominal pain or discomfort at least 3 days a month in the last 3 months. In addition, at least two of the following criteria needed to be fulfilled: (i) improvement with defecation; (ii) a change in the frequency of stools; and (iii) a change in the appearance of stools.

### Statistical analysis

Data were analyzed using statistical software SPSS (version 20, SPSS Science, USA) and described in the form of mean and standard deviation for quantitative data and as frequencies and proportions (%) for qualitative data. Student's *t*‐test was used to examine the difference between the means of two independent groups. Chi‐square test was used to determine the association between qualitative variables. *P* values <0.05 were considered significant.

## Results

**Figure 1:**
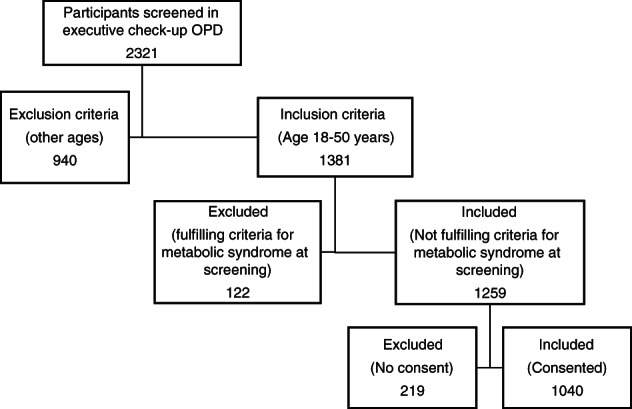
Flowchart of participant enrolment.

As shown in the flowchart (Fig. 1), we included 1040 participants. The mean age of the participants was 37.3 ± 7.1 years, and the mean weight was 72.2 ± 12.7 kg (range 40–140 kg). The ratio of males to females was 3:1 (801 males and 239 females) with maximum participants (45.5%) in the age group of 31–40 years. The mean BMI of males and females in our study sample was comparable (26.0 ± 3.8 kg/m^2^ [range 16–45 kg/m^2^] and 26.7 ± 4.5 kg/m^2^ [range 16–43 kg/m^2^], respectively).

### Metabolic syndrome

The sociodemographic characteristics of participants with and without metabolic syndrome are depicted in Table 1. The prevalence of metabolic syndrome in our study was found to be 307 of 1040 (29.5%). It was found that with increasing age, the prevalence of metabolic syndrome also increased, and this association was significant (*P* < 0.001). Also, metabolic syndrome was significantly associated with BMI (*P* < 0.001). Central obesity (waist circumference ≥90 cm in males and ≥80 cm in females) and reduced HDLC levels (<40 mg/dL in males and <50 mg/dL in females) were the two most prevalent criteria of metabolic syndrome (259 of 1040 [24.9%] and 268 of 1040 [25.8%] respectively). With an increase in BMI, the prevalence of metabolic syndrome increased, being highest in the overweight and obese category ([24.8% among overweight participants with BMI 23–27.5 kg/m^2^ and 50.5% among obese participants with BMI >27.5 kg/m^2^, respectively], data not shown].

**Table 1:** Comparison of sociodemographic characteristics between groups (*n* = 1040)

	Metabolic syndrome			
Characteristic	Present (307)	Absent (733)	Total	*P*
Age (years)				
18–30	33 (16.6)	166 (83.4)	199	**<0.001**
31–40	129 (27.3)	344 (72.7)	473	
41–50	145 (39.4)	223 (60.6)	368	
Sex				
Male	237 (29.6)	564 (70.4)	801	0.929
Female	70 (29.3)	169 (70.7)	239	
Education				
Up to 12th grade	44 (14.3)	82 (11.2)	126	0.187
Graduate/postgraduate	263 (28.8)	651 (71.2)	914	
Residence				
Rural	0 (0.0)	3 (100)	3	0.262
Urban	307 (29.6)	730 (70.4)	1037	
Occupation				
Service	277 (29.3)	669 (70.7)	946	0.593
Unemployed	30 (31.9)	64 (68.1)	94	

*Note*: Bold values indicate significant *P* values.

**Figure 2:**
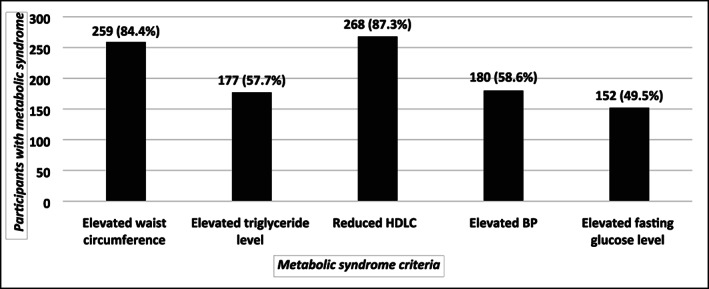
Distribution of the five metabolic syndrome‐defining criteria among participants with metabolic syndrome (*n* = 307).

Figure 2 shows the distribution of the five metabolic syndrome‐defining criteria among participants with metabolic syndrome. As can be seen, reduced HDL cholesterol (87.3%) and elevated waist circumference (84.4%) were the two most prevalent parameters found in those diagnosed with metabolic syndrome.

### IBS and metabolic syndrome components

**Figure 3:**
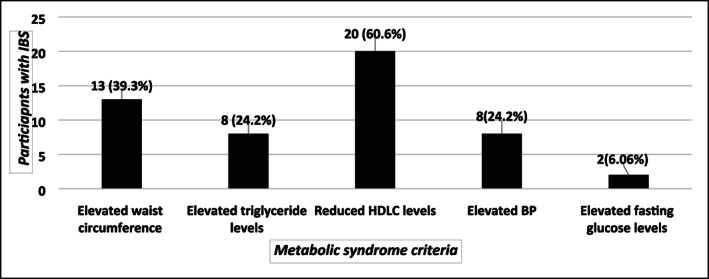
Distribution of the five metabolic syndrome‐defining criteria among participants with irritable bowel syndrome (*n* = 33).

By using the Rome III criteria, IBS was diagnosed in 33 of 1040 participants (3.2%). As seen in Table 2, there was no statistically significant difference between the prevalence of IBS in participants with and without metabolic syndrome (5 of 307 [1.6%] *vs* 28 of 733 [3.8%] respectively, *P* = 0.06). Table 3 shows the comparison of various characteristics including the mean values of metabolic syndrome‐defining parameters between individuals with and without IBS. The difference in the mean values was statistically significant for elevated waist circumference and elevated fasting glucose level (*P* = 0.011 and *P* < 0.001 respectively). Also, it can be seen that when compared with the non‐IBS group, those with IBS were significantly heavier (*P* = 0.009) and also had a greater BMI (*P* = 0.002). Figure 3 shows the distribution of metabolic syndrome‐defining parameters among participants diagnosed with IBS. It can be seen that reduced HDLC level (60.6%) and elevated waist circumference (39.3%) were the most prevalent.

**Table 2:** Association between metabolic syndrome and irritable bowel syndrome

	Metabolic syndrome		
Irritable bowel syndrome	Yes (*n* = 307)	No (*n* = 733)	*P* value
Yes	5 (1.6)	28 (3.8)	0.06
No	302 (98.4)	705 (96.2)	

**Table 3:** Comparison of characteristics between patients with and without irritable bowel syndrome (IBS)

Characteristic	IBS (*n* = 33) Mean ± SD	No IBS (*n* = 1007) Mean ± SD	*P* value
Age (years)	37.39 ± 7.05	35.45 ± 7.13	0.135
Sex (males %)	75.8	77.1	0.861
Weight (kg)	72.41 ± 12.72	67.15 ± 10.73	**0.009**
Waist circumference (cm)	88.76 ± 8.88	85.18 ± 7.53	**0.011**
BMI (kg/m^2^)	26.25 ± 3.94	24.23 ± 3.37	**0.002**
SBP (mm Hg)	118.33 ± 13.0	117.27 ± 8.75	0.508
DBP (mm Hg)	75.08 ± 7.84	73.33 ± 5.40	0.204
TG level (mg/dL)	128.86 ± 72.68	117.61 ± 54.79	0.378
HDL cholesterol (mg/dL)	39.62 ± 9.15	42.04 ± 11.38	0.235
Fasting glucose (mg/dL)	96.08 ± 25.37	89.06 ± 7.42	**<0.001**

*Note*: Bold values indicate significant *P* values.

DBP, diastolic blood pressure; SBP, systolic blood pressure; TG, triglycerides.

## Discussion

Proper bowel function has always been considered as a sign of generalized well‐being. The biopsychosocial conceptual model of diseases by Engel followed by developments in the field of neurogastroenterology has come a long way to improve our understanding of gut–brain dysfunction and its biochemical effects.^19^ Investigations into the gut microbiome have revealed interesting associations with the host metabolism substantiating its possible role in cardiometabolic disorders.^20^, ^21^ This paved the way for various studies exploring the association between functional GI disorders and metabolic syndrome phenotypes.

In this cross‐sectional study of individuals coming for health screening at a tertiary care center, we examined the association between newly diagnosed metabolic syndrome and IBS. Although the prevalence of metabolic syndrome and IBS is comparable to previous studies, we did not find any association between the two.

The prevalence of metabolic syndrome in our study of 29.5% is lower than that reported from South India (33%)^22^ and North India (40.9%).^23^ This can be explained by the fact that we excluded patients already diagnosed with hypertension, diabetes mellitus, or dyslipidemia. Thus, the prevalence in the present study reflects newly diagnosed cases of metabolic syndrome only.^24^ In our study, central obesity and reduced HDL cholesterol were most prevalent (24.9% and 25.8% respectively). Similar findings have been reported by Mohan et al. (CURES study) and Gupta et al.^25^, ^26^

The prevalence of IBS in our study was 3.2% using Rome III criteria. This is lower as compared with previous studies like Ghoshal et al. (4.2%),^4^ Makharia et al. (4%),^27^ and Perveen et al. (7.7%).^28^ Makharia found that the highest prevalence was in the age group of 51–60 years.^27^ A lower prevalence in the present study could be because we excluded participants above the age of 50 years. IBS is a symptom‐based diagnosis. The sociocultural differences in describing the symptoms could explain the different prevalence rates across various countries. To address this, the second Asian consensus on IBS has suggested some modifications in the Rome criteria for the Asian population.^29^

We used the Rome III version as the Rome IV version was not published when our study was conducted. Oka et al. have reported that the prevalence of IBS was substantially lower with Rome IV criteria, suggesting that these might be less suitable for population‐based epidemiological studies.^30^

We did not find any association between metabolic syndrome and IBS, as has been reported by various studies.^1^, ^8^, ^16^ Apart from the obvious reason that the null hypothesis is true, there could be other possible explanations. We know that IBS is not the only functional bowel disorder, and bowel dysfunction, if associated with metabolic syndrome, could present itself in myriad ways. Also, the surreptitious character of these entities, leading to an inherent antecedent‐consequent bias, is a unique limitation of cross‐sectional studies. In the present study, the patients with already‐known components of metabolic syndrome were carefully excluded to nullify the confounding effects of change in lifestyle, diet, and drugs such as metformin, statins, and anti‐hypertensives on the gut function. This unique participant enrolment strategy could explain why other studies have found a positive association between these conditions. Furthermore, we did not use the translated version of the Enhanced Asian Rome III questionnaire (EAR3Q), a cultural adaptation of the Rome III questionnaire,^31^ which could have influenced the prevalence of IBS in the present study, thereby influencing the result.

However, we did find that there were significantly higher mean values of some of the metabolic syndrome‐defining parameters in participants diagnosed with IBS as compared with those without IBS. We found higher mean values of waist circumference, weight, BMI, and fasting glucose and lower mean HDLC in participants with IBS. The mean elevated fasting glucose level was found to have a significant association with IBS in our study, similar to the finding by Gulcan et al.^10^ We also found an association between waist circumference and IBS akin to the finding reported by Nuaman.^13^ The significant association between higher BMI and IBS in our study is in line with the finding from the Egyptian study.^32^ Also, we found a significant association between increased weight and IBS, similar to that reported by Blakely^33^ and Cholongitas.^8^

IBS and metabolic syndrome are disorders that are complex, multifactorial, deceptive in their presentation, and having far‐reaching consequences. Negative results from this study, by no means, put a lid on the topic of the relationship between metabolic syndrome and functional GI disorders, including IBS. We hope that this study will encourage researchers to conduct well‐designed longitudinal cohort studies in the future, which may help us understand the association between gut function and metabolic phenotypes.

## Limitations

The fact that our sample size was calculated based on the prevalence of metabolic syndrome rather than the prevalence of IBS among individuals with metabolic syndrome, could have resulted in a type II error, thus overtly influencing our conclusion of there being no association between IBS and metabolic syndrome. Another limitation is the use of Rome‐foundation‐approved (albeit non‐validated) Marathi translation of the Rome III questionnaire.

## Conclusion

The prevalence of metabolic syndrome was found to be high in our predominantly urban sample. The prevalence of IBS was low based on Rome III criteria. We did not find a significant association between metabolic syndrome and IBS.
